# AMPK-α1 or AMPK-α2 Deletion in Smooth Muscles Does Not Affect the Hypoxic Ventilatory Response or Systemic Arterial Blood Pressure Regulation During Hypoxia

**DOI:** 10.3389/fphys.2018.00655

**Published:** 2018-06-06

**Authors:** Sandy MacMillan, A. Mark Evans

**Affiliations:** Centre for Discovery Brain Sciences and Centre for Cardiovascular Science, College of Medicine and Veterinary Medicine, University of Edinburgh, Edinburgh, United Kingdom

**Keywords:** AMPK, hypoxia, ventilation, blood pressure, smooth muscle

## Abstract

The hypoxic ventilatory response (HVR) is markedly attenuated by AMPK-α1 deletion conditional on the expression of Cre-recombinase in tyrosine hydroxylase (TH) expressing cells, precipitating marked increases in apnea frequency and duration. It was concluded that ventilatory dysfunction caused by AMPK deficiency was driven by neurogenic mechanisms. However, TH is transiently expressed in other cell types during development, and it is evident that central respiratory depression can also be triggered by myogenic mechanisms that impact blood supply to the brain. We therefore assessed the effect on the HVR and systemic arterial blood pressure of AMPK deletion in vascular smooth muscles. There was no difference in minute ventilation during normoxia. However, increases in minute ventilation during severe hypoxia (8% O_2_) were, if affected at all, augmented by AMPK-α1 and AMPK-α2 deletion in smooth muscles; despite the fact that hypoxia (8% O_2_) evoked falls in arterial *Sp*O_2_ comparable with controls. Surprisingly, these mice exhibited no difference in systolic, diastolic or mean arterial blood pressure during normoxia or hypoxia. We conclude that neither AMPK-α1 nor AMPK-α2 are required in smooth muscle for the regulation of systemic arterial blood pressure during hypoxia, and that AMPK-α1 deficiency does not impact the HVR by myogenic mechanisms.

## Introduction

The AMP-activated protein kinase (AMPK) is a cellular energy sensor that maintains cell-autonomous energy homeostasis. From its 2 α (catalytic), 2 β and 3 γ (regulatory) subunits 12 AMPK heterotrimers may be formed, each harboring different sensitivities to activation by increases in cellular AMP and ADP, and the capacity to directly phosphorylate and thus regulate different targets (Ross et al., 2016). AMPK is coupled to mitochondrial oxidative phosphorylation by two discrete albeit cooperative pathways, involving liver kinase B1 (LKB1) and changes in the cellular AMP:ATP and ADP:ATP ratios. Binding of AMP to the AMPK γ subunit increases activity 10-fold by allosteric activation alone, while AMP or ADP binding delivers increases in LKB1-dependent phosphorylation and reductions in dephosphorylation of Thr172 on the α subunit that confer 100-fold further activation. All of these effects are inhibited by ATP (Gowans et al., 2013). LKB1 is, therefore, the principal pathway for AMPK activation during metabolic stresses such as hypoxia. However, there are alternative Ca^2+^-dependent pathways to AMPK activation that are governed by the calmodulin-dependent protein kinase CaMKK2, which delivers increases in Thr172 phosphorylation and thus AMPK activation independent of changes in cellular AM(D)P:ATP ratios.

Classically AMPK regulates cell-autonomous pathways of energy supply by phosphorylating targets that switch off non-essential anabolic processes that consume ATP and switch on catabolic pathways that generate ATP, thereby compensating for deficits in ATP supply or availability (Ross et al., 2016). Recently, however, we demonstrated (Mahmoud et al., 2016) that the role of AMPK in metabolic homeostasis is not limited to such cell autonomous pathways, but extends to the hypoxic ventilatory response (HVR) (Teppema and Dahan, 2010; Wilson and Teppema, 2016) and thus O_2_ and energy (ATP) supply to the body as a whole. In doing so AMPK acts to oppose central respiratory depression during hypoxia and thus resists hypoventilation and apnea.

However, the HVR could well be affected by AMPK deficiency in cell systems other than catecholaminergic neurons due to off-target AMPK deletion through “leakage” of Cre beyond those cells targeted by the conditional deletion strategy we employed. This possibility is highlighted by the fact that transient developmental expression of TH occurs in disparate cell groups that do not express TH in the adult (Lindeberg et al., 2004), including, for example, a subset of heart wall cells. Therefore, there remains the possibility that AMPK deficiency might somehow affect ventilatory control mechanisms through myogenic rather than neurogenic mechanisms. This is evident from the fact that systemic arteries dilate in response to tissue hypoxemia in order to match local perfusion to local metabolism (Roy and Sherrington, 1890), and evidence suggests a role for AMPK in this response (Goirand et al., 2007; Schneider et al., 2015). With “leakage” of Cre, off-target deletion of AMPK in arterial myocytes could attenuate arterial dilation and thus impact O_2_ supply to the brain during hypoxia and the HVR. This possibility is highlighted by the fact that respiratory failure can be triggered through the Cushing reflex consequent to marked increases in blood pressure (Guyenet, 2000; Paton et al., 2009).

We therefore assessed the impact of AMPK deficiency in smooth muscles on blood pressure control and the HVR. Our data show that AMPK does not contribute to the HVR or blood pressure control during hypoxia through myogenic mechanisms.

## Materials and Methods

Experiments were performed in accordance with the regulations of the United Kingdom Animals (Scientific Procedures) Act of 1986. All studies and breeding were approved by the University of Edinburgh and performed under UK Home Office project licenses. Both male and female mice were used, and all were on a C57/Bl6 background. Numbers of mice (≥3 per measure) used are indicated for each experiment. Global, dual knockout of the genes encoding AMPK-α1 (*Prkaa1*) and AMPK-α2 (*Prkaa2*) is embryonic lethal. We therefore employed conditional deletion of the genes for the AMPK-α1 or AMPK-α2 subunits, using mice in which the sequence encoding the catalytic site of either or both of the α subunits was flanked by loxP sequences (Lantier et al., 2014). To direct AMPK deletion to cells of the smooth muscle lineage, these were crossed with mice in which Cre recombinase was under the control of the transgelin (smooth muscle protein 22α) promoter (The Jackson Laboratory, Bar Harbor, ME, United States [stock number 017491, Tg(Tagln-cre)1Her/J in C57BL/6:129SJL background]). The presence of wild-type or floxed alleles and CRE recombinase were detected by PCR. We used four primers for Cre (Transgene 5′-GCGGTCTGGCAGTAAAAACTATC-3′ and 5′-GTGAAACAGCATTGCTGTCACTT-3′; internal positive control 5′-CTAGGCCACAGAATTGAAAGATCT-3′ and 5′-GTAGGTGGAATTTCTAGCATCATCC-3′; expected size WT = 324 bp, Cre = 100 bp). Two primers were used for each AMPK catalytic subunit: α1-forward 5′-TATTGCTGCCATTAGGCTAC-3′, α1-reverse: 5′-GACCTGACAGAATAGGATATGCCCAACCTC-3′ (WT = 588 bp, Floxed = 682 bp); α2-forward 5′-GCTTAGCACGTTACCCTGGATGG-3′, α2-reverse: 5′-GTTATCAGCCCAACTAATTACAC-3′ (WT = 204 bp, Floxed = 250 bp). 10 μl samples were run on 2% agarose gels with 0.01% v/v SYBR^®^Safe DNA Gel Stain (Invitrogen) in TBE buffer against a 100 bp DNA ladder (GeneRuler^TM^, Fermentas) using a Model 200/2.0 Power Supply (Bio-Rad). Gels were imaged using a Genius Bio Imaging System and GeneSnap software (Syngene).

### End-Point and Quantitative RT-PCR

For qPCR analysis, 2 μl of cDNA in RNase free water was made up to 20 μl with 10 μl FastStart Universal SYBR Green Master (ROX, Roche), 6.4 μl Ultra Pure Water (SIGMA) and forward and reverse primers for AMPK-α1 and AMPK-α2. The sample was then centrifuged and 20 μl added to a MicroAmpTM Fast Optical 96-Well Reaction Plate (Greiner bio-one), the reaction plate sealed with an optical adhesive cover (Applied Biosystems) and the plate centrifuged. The reaction was then run on a sequence detection system (Applied Biosystems) using AmpliTaq Fast DNA Polymerase, with a 2 min initial step at 50°C, followed by a 10 min step at 95°C, then 40x 15 s steps at 95°C. This was followed by a dissociation stage with 15 s at 95°C, 20 s at 60°C, and 15 s at 95°C. Negative controls included control cell aspirants for which no reverse transcriptase was added, and aspiration of extracellular medium and PCR controls. None of the controls produced any detectable amplicon, ruling out genomic or other contamination.

### Plethysmography

As described previously (Mahmoud et al., 2016), we used a whole-body unrestrained plethysmograph, incorporating a Halcyon^TM^ low noise pneumatochograph (Buxco Research Systems, United Kingdom) coupled to FinePointe acquisition and analysis software (Data Science International, United States). Following acclimation and baseline measurements (awake but quiet, undisturbed periods of breathing) under normoxia (room air), mice were exposed to hypoxia (8% O_2_, with 0.05% CO_2_, balanced with N_2_) for 10 min. The FinePointe software automatically calculated the respiratory parameters assessed after application of exclusion criteria due to non-ventilatory artifacts (movement, sniffing, etc.). Data were acquired as 2 s averages and 2 to 4 data points of undisturbed breathing were selected for each time point of the HVR. Apneas were defined as a period of cessation of breathing that was greater than the average duration, including interval, of two successive breaths (∼600 ms) of control mice during normoxia with a detection threshold of 0.25 mmHg (SD of noise).

### Blood Pressure Measurements

Blood pressure was recorded using a CODA non-invasive blood pressure system (Kent Scientific, United States). Following five consecutive days of habituation “training,” mice were restrained using a pre-warmed mouse holder and placed on a warming platform (Kent Scientific, United States). Resting blood pressure was measured 32 times per session and only stable readings throughout each session were considered for analysis. On separate days hypoxic blood pressure was measured using the following protocol: 13 cycles of normoxia, 16 cycles of hypoxia (8% O_2_, with 0.05% CO_2_, balanced with N_2_; approximately 10 min) and finally eight cycles of recovery (approximately 5 min). Hypoxic gas originating from a pre-mixed gas cylinder (BOC, United Kingdom) was delivered at a flow rate of 2 L/min via a head cone that inserts into the mouse holder.

### Measurement of Arterial Oxygen Saturation and Heart Rate

Arterial *Sp*O_2_ measurements of mice were obtained using the MouseSTAT^TM^ Pulse Oximeter (Kent Scientific, United States) and an infrared Y-style tail sensor at a sampling rate of 0.5 Hz. As above, mice were restrained using a pre-warmed mouse holder and placed on a warming platform (Kent Scientific, United States). Following 5 min of acclimation, baseline *Sp*O_2_ and heart rates were measured for 2 min immediately preceding a 10-min period of hypoxia (8% O_2_, with 0.05% CO_2_, balanced with N_2_), followed by 3–5 min of recovery. Hypoxic gas originating from a pre-mixed gas cylinder (BOC, United Kingdom) was delivered at a flow rate of 2 L/min via a head cone that inserts into the mouse holder.

### Statistical Analysis

Statistical comparison was completed using GraphPad Prism 6 for the following: plethysmography, two-way ANOVA with Bonferroni multiple comparison’s test; blood pressure, one-way ANOVA with Bonferroni multiple comparison’s test, *Sp*O_2_, two-way ANOVA with Bonferroni multiple comparison’s test. *p* < 0.05 was considered significant.

## Results

We investigated the possibility that AMPK-α1 or AMPK-α2 subunits might support myogenic responses to hypoxia and thus impact the HVR and blood pressure control. To achieve this goal we developed mice with conditional deletion in smooth muscles of the gene encoding AMPK-α1 (*Prkaa1*) and AMPK-α2 (*Prkaa2*), respectively. Critical exons of the AMPK-α1 and -α2 subunit genes were flanked by loxP sequences (Lantier et al., 2014), and each floxed mouse line was crossed with mice expressing Cre recombinase under the control of the transgelin promoter (smooth muscle protein 22α) (El-Bizri et al., 2008). Previous studies have shown that transgelin-Cre mice do not exhibit Cre expression in endothelial cells, and therefore provide for selective gene deletion in smooth muscle versus endothelial cells (**Supplementary Figure S1**). It should be noted, however, that transient developmental expression of transgelin has been observed in atrial and ventricular myocytes. Consequently, genomic recombination will also occur in these cells despite the fact that they do not express transgelin in the adult (El-Bizri et al., 2008). Therefore, while imperfect, the use of transgelin-Cre mice provided us with the necessary level of specificity to determine the role of smooth muscle AMPK in myogenic responses to hypoxia that impact the HVR and blood pressure control.

### The Hypoxic Ventilatory Response Is Not Attenuated by AMPK-α1 or AMPK-α2 Deficiency in Smooth Muscles

**FIGURE 1:**
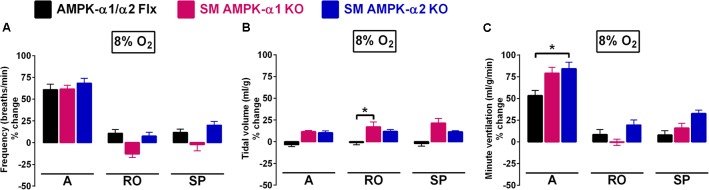
The hypoxic ventilatory response (HVR) is not attenuated by conditional deletion of AMPK-α1 and AMPK-α2 subunits in smooth muscles. Bar charts show mean ± SEM for changes in **(A)** respiratory frequency, **(B)** tidal volume, and **(C)** minute ventilation for AMPK-α1/α2 floxed mice (Flx; black, *n* = 12), and for mice with AMPK-α1 (*n* = 4; magenta) and AMPK-α2 (*n* = 4; blue) deletion in smooth muscles (transgelin expressing cells) during the peak of the Augmenting phase (A), after approximately 100s of Roll Off (RO) and the plateau of the Sustained Phase (SP) of the response to hypoxia; ^∗^*p* < 0.05.

Mice with conditional deletion of AMPK-α1 or AMPK-α2 subunits in smooth muscles exhibited no obvious phenotype and no ventilatory dysfunction or deficiency was evident during normoxia (not shown). Most significantly in the context of the present investigation, we identified no significant attenuation of the HVR relative to controls (AMPK-α1/α2 floxed; **Figure 1** and **Supplementary Figure S2A**). That said, we did observe subtle differences between genotypes.

For smooth muscle AMPK-α1 knockouts alone, there appeared to be an insignificant yet noticeable lowering, relative to controls, of breathing frequency during hypoxia following Roll-Off (≈100 s; -13 ± 4 and 11 ± 4%, respectively) that was maintained for the duration of the Sustained Phase (2–5 min; -2 ± 7 and 12 ± 4%, respectively) of the HVR (**Figure 1A**). By contrast, in both AMPK-α1 and AMPK-α2 knockouts larger increases in tidal volume were observed when compared to controls (**Figure 1B** and **Table 1**), which reached significance for AMPK-α1 knockouts, but only at the peak of Roll-Off (17 ± 6 versus -1 ± 3%, respectively; *p* < 0.05). Increases in minute ventilation during hypoxia also showed a tendency to be greater for AMPK-α1 and AMPK-α2 knockouts than for controls (AMPK-α1/α2 floxed). However this only reached significance during the initial Augmenting Phase of the HVR (**Figure 1C**; *p* < 0.05), which primarily results from increases in carotid body afferent input responses (Day and Wilson, 2007; Teppema and Dahan, 2010; Wilson and Teppema, 2016).

**Table 1:** Means ± SEM of percentage changes in breathing frequency, tidal volume, and minute ventilation and values for apnea frequency, apnea duration and apnea duration index during exposures to hypoxia (8%O_2_) across all genotypes.

Genotype	Frequency	Tidal volume	Minute ventilation	Apnea frequency (min^-1^)	Apnea duration (ms)	Apnea duration index
AMPK-α1/α2 floxed	A 61 ± 7%	A -3 ± 2%	A 53 ± 6%	3.0 ± 0.5	936 ± 40	2.9 ± 0.5
	RO 11 ± 4%	RO -1 ± 3%	RO 8 ± 6%			
	SP 12 ± 4%	SP -2 ± 3%	SP 8 ± 5%			
SM AMPK-α1 knockouts	A 62 ± 4%	A 12 ± 1%	A 79 ± 7%	3.4 ± 1	1021 ± 86	3.4 ± 1
	RO -13 ± 4%	RO 17 ± 6%	RO -1 ± 4%			
	SP -2 ± 7%	SP 22 ± 5%	SP 16 ± 5%			
SM AMPK-α2 knockouts	A 68 ± 6%	A 10 ± 2%	A 84 ± 7%	2.4 ± 0.9	900 ± 90	2.3 ± 1
	RO 7 ± 5%	RO 12 ± 2%	RO 19 ± 6%			
	SP 20 ± 4%	SP 11 ± 1%	SP 33 ± 4%			

*A, Augmenting Phase; RO, Roll-Off; SP, Sustained Phase*.

**FIGURE 2:**
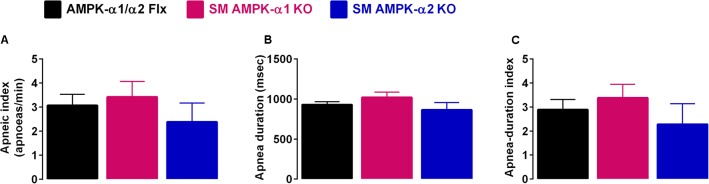
Deletion of AMPK-α1 and AMPK-α2 subunits in smooth muscles does not affect apnea frequency or duration during hypoxia. Bar charts show mean ± SEM for the **(A)** apneic index (per minute), **(B)** apnea duration, and **(C)** apnea duration index (frequency × duration) of AMPK-α1/α2 floxed mice (Flx; black, *n* = 12), and for mice with AMPK-α1 (*n* = 4; magenta) and AMPK-α2 (*n* = 4; blue) deletion in smooth muscles (transgelin expressing cells).

Perhaps most striking was the fact that deletion of neither the AMPK-α1 nor AMPK-α2 catalytic subunits in smooth muscles had any discernible effect on hypoxia-evoked apneas (**Figure 2**, **Table 1**, and **Supplementary Figure S2B**), when compared to controls (AMPK-α1/α2 floxed). This is evident from the fact that there was no difference in either the apnea frequency (**Figure 2A**), duration (**Figure 2B**), or apnea duration index (**Figure 2C**) in mice lacking AMPK-α1 or AMPK-α2 subunits in smooth muscles.

The aforementioned findings are in complete contrast to our observations on mice with AMPK-α1 deletion in catecholaminergic neurons (driven by TH-Cre) which exhibited marked attenuation of the HVR and pronounced increases in apnea frequency, duration and apnea duration index (Mahmoud et al., 2016).

### Cardiovascular Responses to Hypoxia Remain Unaltered Following Deletion of AMPK-α1 and AMPK-α2 in Smooth Muscle Cells

**FIGURE 3:**
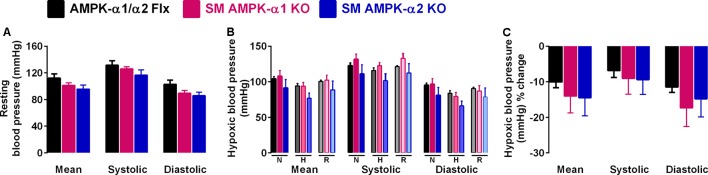
Deletion of AMPK-α1 and AMPK-α2 subunits in smooth muscles has no effect on systemic arterial blood pressure during normoxia or hypoxia. **(A)** Bar charts show (mean ± SEM) the mean, systolic and diastolic blood pressure of control mice [AMPK-α1/α2 floxed (Flx); *n* = 4; black] and mice with AMPK-α1 (*n* = 4; magenta) and AMPK-α2 (*n* = 4; blue) deletion in smooth muscles (transgelin expressing cells). **(B)** Bar charts show the same measures of blood pressure during normoxia (N), after 10 min to hypoxia (H; 8% O_2_) and following recovery to normoxia (R). **(C)** Bar charts show the percentage change in blood pressure during hypoxia.

We next assessed the effect of AMPK deficiency on cardiovascular function during hypoxia by monitoring systemic blood pressure during hypoxia. Deleting either AMPK-α1 or AMPK-α2 subunits in smooth muscle cells had little or no effect on resting blood pressure (**Figure 3A** and **Supplementary Figure S2C**). Furthermore neither smooth muscle AMPK-α1 nor -α2 deficiency affected hypoxia-evoked falls in systemic blood pressure in mice (**Figures 3B,C**, **Table 2**, and **Supplementary Figures S2Cii,iii**), which were comparable to the range of responses reported previously (Campen et al., 2005; Mahmoud et al., 2016).

**Table 2:** Means ± SEM of values obtained by tail cuff blood pressure measurements and percentage changes in arterial *Sp*O_2_ before, during, and after exposures to hypoxia (8%O_2_) across all genotypes.

Genotype	Resting BP	(mmHg)	Mean BP	(mmHg)	Sys BP	(mmHg)	Dias BP	(mmHg)	*Sp*O_2_
AMPK-α1/α2 floxed	M 112 ± 6	N 104 ± 3	N 123 ± 4	N 95 ± 3	N 89 ± 5%
	Sys 131 ± 7	H 94 ± 3.5	H 116 ± 3.5	H 84 ± 4	H 69 ± 5%
	Dias 103 ± 6	R 100 ± 2	R 121 ± 1	R 91 ± 2	R 92 ± 2%
AMPK-α1 knockouts	M 101 ± 4	N 108 ± 8	N 132 ± 7.5	N 97 ± 8	N 96 ± 2%
	Sys 126 ± 3	H 94 ± 5	H 123 ± 5	H 79 ± 6	H 75 ± 4%
	Dias 89 ± 4	R 89 ± 12	R 133 ± 7	R 87 ± 7.5	R 94 ± 1%
AMPK-α2 knockouts	M 96 ± 6	N 91 ± 12	N111 ± 13	N 81 ± 11	N 97 ± 0.5%
	Sys 117 ± 8	H 77 ± 7	H 102 ± 9.5	H 66 ± 6.5	H 71 ± 3%
	Dias 86 ± 5	R 102 ± 7	R 112 ± 13	R 79 ± 12	R 92 ± 2%

*M, mean blood pressure; Sys, systolic blood pressure; Dias, diastolic blood pressure; N, normoxia (room air); H, hypoxia (8% O_2_); R, recovery (room air)*.

**FIGURE 4:**
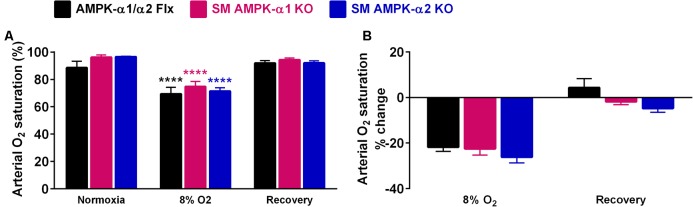
Comparable falls in arterial oxygen saturation were evoked in all genotypes during hypoxia. **(A)** Bar charts show mean ± SEM for the arterial oxygen saturation (*Sp*O_2_) during normoxia, after 10 min of hypoxia (8% O_2_) and following recovery to normoxia. **(B)** Percentage change in *Sp*O_2_. AMPK-α1/α2 floxed (Flx; black, *n* = 3), smooth muscle AMPK-α1 KO (magenta, *n* = 4) and smooth muscle AMPK-α2 KO (blue, *n* = 4) mice; ^∗∗∗∗^*p* < 0.001.

That comparable levels of arterial hypoxia were achieved in all mice was confirmed by pulse oximetry, which demonstrated significant and similar falls in *Sp*O_2_ for all genotypes (**Figure 4** and **Supplementary Figure S2D**, *p* < 0.0001). Moreover there was no difference with respect to post-hypoxic recovery of arterial *Sp*O_2_ upon return to room air.

## Discussion

The present investigation demonstrates that the HVR is, if anything, slightly augmented rather than attenuated by AMPK-α1 or AMPK-α2 deficiency in smooth muscles (driven by transgelin-Cre). This outcome is in marked contrast to our previously reported finding that AMPK-α1 deficiency in catecholaminergic neurons (driven by TH-Cre) markedly attenuates the HVR and precipitates hypoventilation and apnea during hypoxia (Mahmoud et al., 2016). Therefore, outcomes are consistent with our previously proposed model of central oxygen-sensing by catecholaminergic neurons of the brainstem respiratory network. Briefly, we proposed that these neurons deliver increases in respiratory drive during hypoxia in a manner supported by AMPK heterotrimers incorporating the catalytic α1 subunit, the activity of which we hypothesized to be determined by integration of local hypoxic stress at the brainstem with carotid body afferent input responses that provide an index of peripheral hypoxia. Notably, natural selection in high-altitude (Andean) populations has led to single nucleotide polymorphisms in PRKAA1 (Bigham et al., 2014).

The significance of our present findings lies in the fact that reduced cerebral arterial dilation could affect O_2_ supply to the brain during hypoxia and thus the HVR through consequent respiratory depression, because the activity of brainstem respiratory networks is ultimately reliant on O_2_ delivery via the vasculature that could be modulated systemically and locally through myogenic responses. Respiratory failure may also be triggered through the Cushing reflex consequent to increases in blood pressure (Guyenet, 2000; Paton et al., 2009), which would be exacerbated by block of arterial dilation consequent to AMPK deletion in arterial myocytes. Such system-specific responses could well be affected by a number of mechanisms through which AMPK has been proposed to regulate myocyte function, rendering outcomes susceptible to off-target AMPK deletion due to “leakage” of Cre beyond those cells targeted by conditional deletion strategies. This was a distinct possibility, given that transient developmental expression of TH occurs in disparate cell groups that do not express TH in the adult (Lindeberg et al., 2004), including, for example, a subset of heart wall cells.

The outcomes of our present investigation indicate that while AMPK has been shown to mediate *ex vivo* arterial dilation in some circumstances (Goirand et al., 2007; Schneider et al., 2015), neither the expression of AMPK-α1 nor AMPK-α2 catalytic subunits in smooth muscles is a pre-requisite for *in vivo* arterial dilation during hypoxia sufficient enough to impact systemic arterial blood pressures. This is evident because we observed little or no difference in peripheral hypoxic vasodilation between genotypes tested here. Moreover, systemic arterial pressures during normoxia and hypoxia were within the typical range previously reported for wild-type mice (Campen et al., 2004). Our findings do not, however, rule out a role for AMPK in maintaining resting vascular tone locally, or in governing organ-/tissue-specific perfusion and oxygen supply. Indeed, studies on other vascular beds have already revealed that AMPK might adjust local perfusion during hypoxia. For example, AMPK may mediate hypoxic pulmonary vasoconstriction (Evans et al., 2005, 2006; Evans, 2006), and thus assist ventilation-perfusion matching by diverting blood from oxygen deprived to oxygen rich areas of the lung (Bradford and Dean, 1894; von Euler and Liljestrand, 1946). Furthermore, evidence suggests that AMPK supports dilation of systemic arteries, such as the aorta and mesenteric arteries, at the level of smooth muscles that may counter tissue hypoxemia (Schneider et al., 2015; Moral-Sanz et al., 2016). Accordingly, AMPK has also been implicated in the regulation of uterine artery reactivity during hypoxia (Skeffington et al., 2015), perhaps linking maternal metabolic and cardiovascular responses during pregnancy and governing oxygen and nutrient supply to the fetus.

The mechanisms involved in systemic arterial dilation during hypoxia include AMPK-dependent activation of SERCA and BK_Ca_ channels in systemic arterial myocytes (Schneider et al., 2015), while AMPK exerts its effects on pulmonary arterial smooth muscle cells, at least in part, through direct phosphorylation and inhibition of the voltage-gated potassium channel K_V_1.5 (Moral-Sanz et al., 2016). Significant to the context of our study, K_V_1.5 availability has been proposed to impact cerebral myogenic responses (Koide et al., 2018), which could equally well be affected by loss of capacity for AMPK-dependent activation of SERCA and BK_Ca_ channels. Either way, our data argue strongly against the possibility that the HVR is influenced by AMPK-dependent mechanisms within smooth muscles that might affect local myogenic responses, irrespective of the tissue- and circulation-specific function they might impact. Nevertheless, it would be interesting to determine whether AMPK contributes to local autoregulatory mechanisms in the cerebral vasculature during hypoxia, even though we find no evidence of a significant contribution to the HVR or peripheral control of systemic blood pressures, which was the focus of this investigation. That said, we cannot rule out the possibility that the outcome of our previous studies using TH-Cre driven AMPK deletion might have been affected through off-target effects at the level of the vascular endothelium, because the conditional deletion strategy used here does not produce Cre expression in endothelial cells (El-Bizri et al., 2008). However, this seems unlikely given the outcomes of our present investigation. Therefore, hypoxic ventilatory dysfunction precipitated by AMPK-α1 deletion conditional on Cre expression in catecholaminergic neurons does not result, in whole or in part, from AMPK-α1 or AMPK-α2 deficiency in smooth muscles and consequent changes in systemic arterial blood pressure during hypoxia.

In short, AMPK likely supports the HVR through neurogenic but not myogenic mechanisms as previously proposed, by supporting increased respiratory drive (Mahmoud et al., 2016) and perhaps functional hyperemia (Bucher et al., 2014), each of which may be coordinated by catecholaminergic neurons of the brainstem cardiorespiratory network.

## Author Contributions

AME and SM wrote the manuscript. AME developed the conditonal *AMPK* knockout mice. AME and SM bred and genotyped the mice, performed plethysmography, blood pressure, and arterial oxygen saturation measurements, and analyzed the data.

## Conflict of Interest Statement

The authors declare that the research was conducted in the absence of any commercial or financial relationships that could be construed as a potential conflict of interest.

## Supplementary Material

FIGURE S1 End-point RT-PCR confirms AMPK deletion. End-point RT-PCR amplicons for transgelin and the catalytic α1 and α2 subunits of AMPK from lungs (right) and primary cultures of pulmonary arterial smooth muscle cells (PASMC, left) obtained from transgelin-Cre, C57BL6 (WT), AMPK-α1 and AMPK-α2 knockout mice.

FIGURE S2 Deletion of AMPK-α1 or AMPK-α2 subunits in smooth muscles does not attenuate the HVR and has no effect on either apnea duration index or systemic arterial blood pressures during hypoxia. Box plots show the median (solid line), 25^th^ to 75^th^ percentiles (boxes), mean (+) and whiskers according to Tukey’s method for: the % change of the ventilatory components of **(Ai)** breathing frequency, **(Aii)** tidal volume, and **(Aiii)** minute ventilation during the initial Augmenting Phase (A), the Roll-Off (RO), and the Sustained Phase (SP) of the hypoxic ventilatory response; **(Bi)** the apneic index (per minute), **(Bii)** apnea durations (in msec) and (Biii) apnea duration index (frequency x duration); Mean, Systolic and Diastolic blood pressures (in mmHg) measured **(Ci)** at rest, **(Cii)** before, during, and after a 10 min hypoxic challenge, and **(Ciii)** as % change during hypoxia; arterial oxygen saturation (in %) during **(Di)** normoxia, 10 min hypoxia, and recovery, and **(Dii)** as % change during hypoxia and recovery. ^∗^*p* < 0.05; ^∗∗∗^*p* < 0.001.
