# Supplementary figures and images for: AMPK-α1 or AMPK-α2 Deletion in Smooth Muscles Does Not Affect the Hypoxic Ventilatory Response or Systemic Arterial Blood Pressure Regulation During Hypoxia
Source: Front Physiol. 2018 Jun 6;9:655. doi: 10.3389/fphys.2018.00655 (PMC5997817; doi:10.3389/fphys.2018.00655)

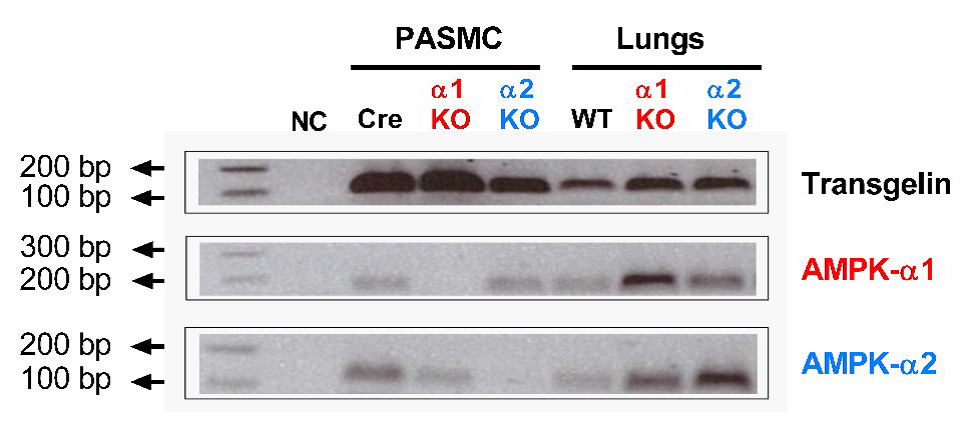

Supplement: FIGURE S1 — End-point RT-PCR confirms AMPK deletion. End-point RT-PCR amplicons for transgelin and the catalytic α1 and α2 subunits of AMPK from lungs (right) and primary cultures of pulmonary arterial smooth muscle cells (PASMC, left) obtained from transgelin-Cre, C57BL6 (WT), AMPK-α1 and AMPK-α2 knockout mice. [file Image_1.TIF]
